# Perspective: cancer vaccines in the era of immune checkpoint blockade

**DOI:** 10.1007/s00335-018-9786-z

**Published:** 2018-11-16

**Authors:** Jonathan Cebon

**Affiliations:** 1 Cancer Immunobiology Laboratory, Olivia Newton-John Cancer Research Institute, Heidelberg, VIC Australia; 2 School of Cancer Medicine, La Trobe University, Bundoora, VIC Australia; 3 Department of Medical Oncology, Austin Health, Heidelberg, VIC Australia

## Abstract

Current excitement about cancer immunotherapy is the result of unprecedented clinical impact from immune checkpoint inhibitors, particularly those that target programmed death (PD)-1 and PD-ligand (L)-1. Numerous other immunotherapeutics are also finding their way into the clinic either alone or in combination, and these have potential applications in many cancer types. Therapeutic cancer vaccines have been a major focus for many pioneers in the field yet have largely failed to live up to expectations as game-changing immunotherapeutics. This, despite decades of focussed efforts that have identified antigens, optimised adjuvants and refined approaches to pre-clinical modelling and clinical monitoring. If antigen-directed immunotherapeutics are to take a place in the anti-cancer therapeutic armamentarium, it will be crucial to understand the potential niche that could be occupied by cancer vaccines that can specifically induce or modify immune response against cancer antigens.

## Clinical impact of immunotherapy

For over a century since Coley’s experiments with bacterial toxins, there have been waves of optimism for anti-cancer immunotherapy, as well as periods of circumspection when clinical trials failed to demonstrate hoped-for impact (Rosenberg et al. 2004). Early approaches with cytokines or cellular products date back to the early 1980s following the identification of immuno-stimulatory cytokines such as interleukin (IL)-2 and the interferons (IFNs). As monotherapies these had limited success for the treatment of cancer, but paved the way for the current era now dominated by the immune checkpoints. This commenced in the early 2000s with the recognition that inhibiting cytotoxic lymphocyte antigen (CTLA)-4 (Egen et al. 2002) and PD-1 (Topalian et al. 2012), and its ligands were powerful new avenues for cancer therapy. A decade earlier researchers largely based at the Ludwig Institute for Cancer Research and at the US National Institute of Health (NIH) first identified human leukocyte antigen (HLA)-restricted peptide cancer rejection antigens (Coulie et al. 1994; Kawakami et al. 1994a, b; Van der Bruggen et al. 1991a). Over the subsequent three decades, numerous clinical trials have been undertaken targeting defined tumour antigens based on the assumption that the ability to enhance antigen-specific immune responses would likely lead to effective anti-cancer therapy reviewed in Cebon et al. (2009).

## Vaccines have not met expectations for clinical impact

It is therefore disappointing when reviewing the early experience, cancer vaccines were found to have relatively little impact despite these approaches being capable of generating human T cell responses, particularly CD8 responses against defined cancer antigens (Rosenberg et al. 2004). Indeed 50 years of trialling therapeutic vaccines in cancer has thus far only yielded a tiny handful of trials which have shown statistically significant impact for vaccines in patients with advanced cancer and only one Food and Drug Administration (FDA) registration: https://www.cancer.gov/about-cancer/treatment/drugs/sipuleucel-T. Despite optimism, many accounts have been anecdotal at best or reflect analyses of retrospective uncontrolled series. With the dominant role of immune checkpoint inhibitors shaping the landscape of cancer immunotherapy and numerous regulatory approvals in just the last 5 years, the question now turns to whether or not antigen-directed therapy has a role to play at all, and if so what it should be.

It is clear that antigen recognition and effector responses against these are prerequisites for anti-cancer responses by the adaptive immune system. It is also clear that vaccines are now effective at inducing or priming responses. For example, our work with NY-ESO-1 vaccination demonstrated the induction of broad-based immune responses involving CD8+ and CD4+ lymphocytes as well as antibodies against multiple epitope derived from the full-length antigen see (Cebon et al. 2010; Jackson et al. 2004) Similarly, there are numerous accounts of vaccines that have induced responses against defined antigens that can be monitored using sensitive in vitro methods (Keilholz et al. 2006; Speiser et al. 2003). So failure of clinical impact is unlikely to reflect an inability to induce immunity. Rather, other processes present obstacles to clinical benefit. These may include barriers within the tumour microenvironment (TME) that the wrong antigens have been chosen as targets for vaccination or that antigen expression is heterogeneous and so eradication of antigen-bearing cells remains inadequate for eradication of the entire tumour.

## The tumour microenvironment

The tumour microenvironment is a complex mixture of cells and immune processes with numerous effectors and regulators. These include CD8+ and CD4+ T-lymphocytes, cells of the innate immune system, myeloid cells and dendritic cells within both the tumour and in regional lymphoid tissue. The many regulators include cytokines which have pro-immune effects such as IFNγ, IL-2, IL-12, molecules which play a homeostatic role such as IL-7 and IL-15 and immunosuppressive cytokines such as transforming growth factor (TGF)β, IL-10 and vascular and endothelial growth factor (VEGF). In addition to cellular immune responses, anti-cancer antibody responses are often generated and have potential impact on anti-cancer immunity (Stockert et al. 1998). Thus, a complex mix of cytokines and cell surface-associated regulatory molecules act to both inhibit and disinhibit immunity. The orchestration of cellular responses is clearly complex involving interplay between a tumour, regional lymphoid tissue and effectors within the circulation. As a result, numerous targets have been identified with therapeutic potential against cancer. Inhibitory or agonistic antibodies directed against these targets are establishing themselves as the most effective clinical tools for manipulating the fine control of anti-cancer immunity.

**Fig. 1:**
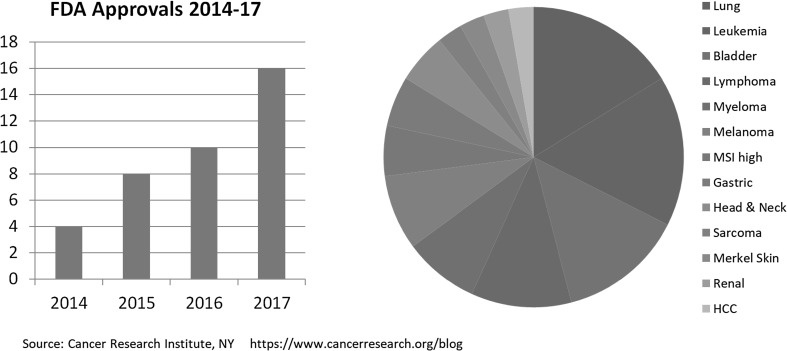
FDA approvals. FDA approvals of immunotherapeutics 2014–2017

Inhibition of the PD-1, PD-L1 axis has been evaluated in a large series of clinical trials and inhibition of this checkpoint alone has demonstrated clinical efficacy in at least 20 different cancer types with a range of responses seen. In some, such as classical Hodgkin’s lymphoma or cancers with microsatellite instability (MSI) response rates are high. In many others, responses are rarer, but nonetheless a finite proportion of tumours respond to PD-1 inhibition monotherapy. Figure 1 shows that the immunotherapy landscape in oncology is rapidly evolving. FDA approvals represent the tip of the iceberg since approvals require high-level clinical evidence, generally randomised phase III clinical trials. Numerous additional agents are currently in earlier phases of clinical development, and phase I and II trials are rapidly establishing a role for many other immunotherapeutics against a wide range of targets and for numerous cancer types. Consequently, it can be expected that indications and approvals will continue at an exponential rate for the foreseeable future.

**Fig. 2:**
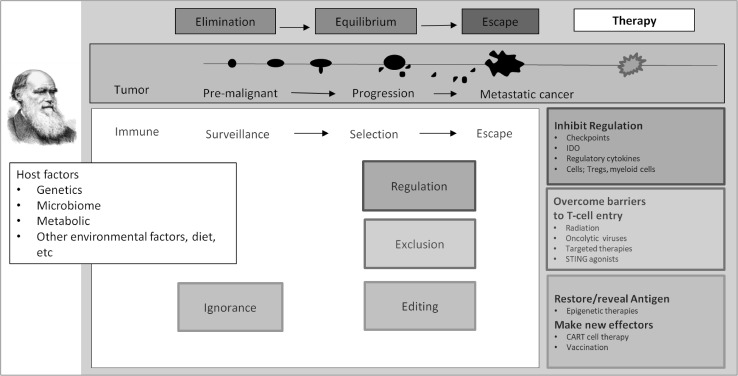
Towards personalised immunotherapy. Cancer and the immune system co-evolve. The three E’s elimination, equilibrium and escape reflect the trajectory of this evolution in which the tumour is sculpted by immuno-editing (Dunn et al. 2004). Escape results from processes that subvert effective anti-cancer immunity; immune regulation, immune exclusion and immune editing. Therapeutic strategies directed at these mechanisms, shown in the corresponding boxes in the right side panels. Cancer vaccination potentially has a role in those situations where the tumour is not recognised by the immune system, either de-novo (immune ignorance) or because antigenic cells have been removed (editing) and so responses against different targets are required. Table 2 elaborates on this in greater detail

The pathways to optimisation including the strategic development of combination approaches are numerous and are beyond the scope here. While the opportunities provided by approved agents is already impressive, additional therapeutics that target other key regulatory mechanisms are following in the pipeline such as indoleamine-2 3-dioxygenase (IDO) (Uyttenhove et al. 2003), lymphocyte-activation gene 3 (LAG3), T cell immunoglobulin and mucin-domain containing-3 (TIM3) and many others (Shin and Ribas 2015). While these have not yet been approved for routine clinical use, randomised trials are likely to yield many additional effective approaches. The development of these opportunities requires a strategic approach as outlined in Table 1. These strategies inevitably point to the need to better personalise therapy. In order to do so the characteristics of the patient, the tumour and the immune response need to be considered and all of these evolve over time (Fig. 2).

**Table 1:** Challenges for optimisation of immunotherapy

1	Identify strategies that will enable additional patient populations to respond: – Who are the non-responders with in responsive tumour types? – What are the best approaches for non-responsive cancers?
2	What are the obstacles: – immune regulation – immune exclusion – immune ignorance – immune escape?
3	What are the best strategies: – Patient selection – Biomarkers – Individualisation of immunotherapy and rational combinations – And importantly is there a role for antigen-specific targeting within this framework?

**Table 2:** Framework for considering a role for vaccination

Tumour characteristics			Likely characteristics of immunity	Potential value of vaccination	Considerations and obstacles
Tumour infiltrating lymphocytes (TILs)		Antigens			
Brisk	Abundant but insufficient to eradicate the cancer	The relationship between abundance and distribution of antigens is not well described. Ag may be homogeneously or heterogeneously distributed. Tumours with Brisk infiltrates such as highly mutated MSI tumours are more likely to have abundant TILs. There are many causes of heterogeneity, e.g. antigen distribution, metabolomic variation, patchy immune editing. While genomically stable tumours with low TMB may be uninflamed, there are many other explanations for a cold phenotype	Established but ineffective	Uncertain; modifying the T cell repertoire and generating new effectors might favourably alter the characteristics of the TME	High likelihood that responsive to single agent ICI, e.g. MSI high tumours
Non-brisk	Excluded or heterogeneous Ag expression		• Probably established • May target heterogeneously expressed antigens	• Induction of responses to a broader range of antigens may overcome heterogeneity • New effectors may modify the characteristics of the TME	Mechanisms driving exclusion or heterogeneity should drive the strategy. Failure of immunity may contribute, however, vaccination is unlikely to overcome mechanisms that generate hostility to T cell effector function
Absent	None		Post-immune:		
			• Heavily regulated TME or • ‘Edited’ tumour bearing antigen-loss variants	• New effectors might kick-start response • Generate a response to new targets	• Hostile TME may be insurmountable • Loss of Ag or HLA may be irreversible
			Pre-immune		
			Naive	Generate/prime immune response for the first time	If low TMB, there may be insufficient suitable antigens

## Cancer vaccine

If you want to know how to vaccinate, you need to know how to immunise (L.J. Old)

Defined neo-antigens recognised by T-lymphocytes are generally considered to be the products of mutated genes. Another class of antigens comprises molecules that are re-expressed in cancer, largely as a result of epigenetic changes. Additionally vaccines can be derived from autologous or allogeneic cells or contain complex mixtures of antigens.

The delivery systems and adjuvants available for vaccine development together with the varieties of antigen preparation are all crucial for optimising the quality of immunity generated by vaccination. These have been reviewed elsewhere (Butterfield 2015).

## Defined antigens

### Products of mutated genes

Genomically unstable cancers such as those with mismatched repair (MMR) deficiencies are frequently responders to PD-1 inhibition (Le et al. 2015). These tumours have long been recognised as having an inflammatory phenotype reflecting an intrinsic antigenicity, and this is associated with better survival (Williams et al. 2018). The high frequency of mutations in these tumours yields an increased frequency of peptide neo-antigens, and these without doubt play a major role in shaping the immunoreactivity. PD1 inhibitors are now approved for the treatment of these tumours—including not only colorectal cancer but a range of other mismatch repair deficient cancers such as cholangiocarcinoma and endometrial carcinoma. There are now many studies showing that tumour mutational burden (TMB) is a biomarker that can identify potentially responsive tumours. In melanoma, recognition that the products of mutated genes are important targets for immune recognition was described early by Lennerz et al. (2005) and subsequently tumours were found to be more likely to respond to cytotoxic T-lymphocyte-associated protein (CTLA4) inhibition if predicted mutational antigens were more abundant (Snyder et al. 2015). Similarly TMB is associated with higher responses in patients with lung cancer (Rizvi et al. 2015). In these cases, mutations burden reflects the mutagenic effects of UV radiation and smoking, respectively.

Such observations have stimulated considerable interest in characterising the peptides derived from mutated genes for antigen-specific vaccination approaches (Capietto et al. 2017). Initial work undertaken in murine sarcomas treated with anti-PD-1 identified potential mutated epitopes through DNA sequencing, validated by showing that lymphocytes specific for the predicted antigens could be found within the tumour microenvironment. Furthermore, vaccines containing long peptides that incorporated the mutations were able to induce tumour rejection in this murine model (Gubin et al. 2014).

The question remains whether vaccination is truly necessary under these circumstances—since the presence of antigen responsive T-lymphocytes within these tumours must indicate that the antigens were immunogenic and had generated a response already. Thus, the failure to eradicate the cancer was not a failure of immune priming. For instance, regulatory mechanisms can be expected to emerge as a homeostatic response to chronic antigenicity. Alternatively, the dominant response may have been directed against antigens that were not present on all tumour cells. This could occur if there was clonal evolution within a tumour. The resultant heterogeneity of antigen expression could allow the emergence of some tumour clones despite the eradication of others. In this scenario, vaccination might be able to induce responses against previously sub-dominant targets.

Several clinical trials have now been reported, whereby antigens predicted from DNA sequencing were used to design vaccines. Sahin et al. (2017) produced “RNA mutanome” vaccines from 13 patients with stage III/IV melanoma. Antigens administered as synthetic RNAs encoding mutated peptides and induced T cell responses against multiple neo-epitopes. Two objective responses were seen following intranodal injections into patients with melanoma. In another small uncontrolled trial, 10 patients were treated with synthetic peptides of up to 20 neo-antigens. These were administered with immunstimulatory molecules: toll-like receptor 3 (TLR3), the MDA5 pattern recognition (MDA5) pattern recognition receptor and Poly-ICLC (Ott et al. 2017). The vaccine was administered after surgical resection of melanoma, and several patients remain disease free.

In another recently reported breast cancer study, adoptively transferred T cells were directed against antigens that were defined by sequencing (Zacharakis et al. 2018). While these studies certainly established proof-of-concept, they were uncontrolled. Randomised trials will be required to demonstrate whether this approach will be superior to treatment with immune checkpoint inhibitors alone.

A major barrier to the viability of this approach as a routine therapy is that creating individual vaccines is time-consuming and likely to be more expensive than ‘off-the-shelf’ solutions. Nonetheless immune checkpoint inhibitory antibodies are expensive even as monotherapies. So the cost of effective combinations may place highly effective personalised vaccines within reach. Perhaps more of a consideration is that tumours need to have a sufficiently high mutational burden to yield antigens with a reliable frequency to enable effectors to be generated. Those which have very low burdens of neo-antigens will be far more challenging for such approaches. Yet these are likely to be the tumours in which spontaneous anti-cancer immunity is rare and so are most likely to benefit from novel approaches. Paradoxically tumours with the highest antigen burden, such as MMR deficient tumours can yield the most potential antigens, yet spontaneous responses will likely be seen anyway, so monotherapy with a PD-1 inhibitor may be all that is required.

### Cancer testis/germline antigens

The first shared human cancer rejection antigens were described in the early 1990s. They include many which are present within germ cells and also re-expressed within cancer (Hofmann et al. 2008; Simpson et al. 2005). Members of this cancer testis (CT) or cancer germline family include MAGE family of antigens, the SPAN-X family of antigens SSX2, SSX3, MAGEC-1, MAGEC-2, ROPN, XAGE and many others (Hofmann et al. 2008). Numerous clinical trials have been performed with these antigens over the years, and these will not be reviewed comprehensively here.

Many are epigenetically repressed as a result of methylation of the antigen promoter. They have highly specific expression as evidenced by their presence in germ cells but rapid disappearance when germ cells mature. Re-emergence in cancer and placenta has meant that these are often highly specific targets for cancer immunotherapy, and a number of vaccines have been developed to target these reviewed in Cebon et al. (2009). MAGE-1 was the first of the antigens recognised by cloned T cells by Boon and colleagues (Traversari et al. 1992; van der Bruggen et al. 1991b), and phase I/II trials have been conducted with MAGE-A antigens. A phase III trial with MAGE-A3 protein was undertaken in patients with fully resected MAGE-A3-expressing melanoma (Dreno et al. 2018). The vaccine had no impact on relapse rates, and thus far none of these vaccines, however, have had clinical impact. This is despite the fact that the antigens are often highly specific to cancer and can be very immunogenic. NY-ESO-1 is another example CT antigen that we have studied in detail using a full-length tumour antigen formulated in ISCOMATRIX™ adjuvant. The vaccine was highly immunogenic in patients with advanced cancers. CD4+, CD8+ T cell and antibody responses were generated in the majority of patients vaccinated. Furthermore, isolated T cells killed antigen expressing targets. Indeed adoptive transfer of NY-ESO-1-specific T-lymphocytes has resulted in occasional dramatic anti-cancer responses, so this antigen appears to be a bona-fide target for antigen-directed approaches (Robbins et al. 2011). Vaccination against NY-ESO-1 has yet to prove effective, however, despite early promise in uncontrolled studies (Lattanzi et al. 2018; Nicholaou et al. 2011).

### Vaccine strategies that do not target-specific defined antigens

There is a considerable literature comprising numerous approaches to vaccination by stimulating immunity to whole tumour. These include tumour lysates, eluted peptides and genetic vaccines derived from tumour nucleic acids. They have been mixed with adjuvants, pulsed onto dendritic cells or introduced into DCs as RNA or in viral vectors (Butterfield 2015; Klein et al. 2011; Neller et al. 2008). None of these approaches have had anything like the clinical impact that has been seen with the immune checkpoint inhibitors.

“In situ” vaccination by injecting immuno-stimulatory agents into the TME can also generate antigen-specific responses and either alone or in combination with other agents has the potential to generate anti-cancer immunity. For example, BCG has been used since the 1970s to stimulate regression in melanoma and in more recent years and is widely applied as standard therapy for early stage bladder cancer (Alexandroff et al. 1999). In an attempt to exploit this approach, we recently undertook an early phase clinical trial with intraregional BCG combined with the CTLA4 inhibitor Ipilimumab (Da Gama Duarte et al. 2018). This trial was discontinued prematurely because of severe auto-immune toxicity in some patients, demonstrating that the approach has the potential to be highly immuno-stimulatory but that some selectivity for cancer may be important in order to constrain toxicity. Along similar lines, an oncogenic virus such as a herpes simplex virus engineers to secrete GM-CSF (Talimogene laherparepvec, T-Vec) has been successfully used to treat local melanoma lesions. In a subset of patients, systemic spreading or extension of the response in uninjected tumours occurred and this agent now has FDA approval for treatment of advanced melanoma (Andtbacka et al. 2015). With the addition of a PD1 inhibitor, further enhancement of ingress of T cells into the tumour microenvironment was seen (Ribas et al. 2017).

### Obstacles to vaccine efficacy

Vaccination approaches utilising molecularly defined antigens have shown evidence of clinical benefit in individual carefully studied patients. Although retrospective uncontrolled reports have been optimistic the only randomised trial thus far to demonstrate impact of vaccination on patient survival has been in prostate cancer using autologous dendritic cells and prostatic acid phosphatase (Kantoff et al. 2010). There are numerous mechanisms by which cancer cells can evade immune recognition and killing, and these include factors that are intrinsic to the tumour cell as well as those in the tumour microenvironment (van der Burg et al. 2016). These may include loss of a single antigen target, intratumoural heterogeneity, down-regulation of HLA expression or loss of specific HLA alleles and immune regulation. Some examples follow in greater detail:

## Heterogeneity

Tumour antigens are often expressed heterogeneously, and this can be a major driver for clonal selection, resistance to therapy and relapse, reviewed in Caswell and Swanton (2017). Studies of CT antigen expression in melanoma have shown that expression is often patchy and may only be evident in a minority of cells (Barrow et al. 2006). This was studied in detail in a trial performed in patients with NY-ESO-1 expressing tumours and screened by immunohistochemistry (NCT00199901). In a previous uncontrolled trial, we saw what was believed to be a clear signal of clinical activity (Nicholaou et al. 2011) and the follow-up study was designed to validate this. Patients with resected stage IIC, III or resected stage IV melanoma were eligible if their tumours were positive by IHC for NY-ESO-1 and randomised to receive either NY-ESO-1 protein formulated in the adjuvant ISCOMATRIX or ISCOMATRIX alone. Patients were then followed to assess clinical relapse-free survival at an 18-month landmark along with other secondary endpoints (submitted). The vaccine was highly effective immunologically and generated NY-ESO-1-specific antibodies, CD4+ and CD8+ T cells. These responses included both the induction of new responses as well as the expansion of pre-existing responses in some cases. Although the vaccine was immunologically potent, there was no impact on survival. To better understand this, biopsies from patients who had relapsed were analysed and using multi-colour immunofluorescence to innumerate cells which doubly expressed NY-ESO-1 antigen and HLA class 1, i.e. those deemed to be immunologically targetable by Ag-specific CD8+ T cells. We found a reduction in these double positive cells. This was either due to loss of HLA or NY-ESO-1. Regardless of which, it appeared that while the vaccine was probably targeting some cells, heterogeneity of antigen expression prevented meaningful clinical impact. Thus HLA loss, as described below, or heterogeneity of antigen expression enabled natural selection of targets that no longer bore targets for immune recognition. This is potentially a major problem for approaches involving the CTAg, since these are seldom uniformly expressed within the cancer. Strategies to counter this could include restricting the selection of patients to those with high levels of Ag expression, or using demethylating agents such as azacytidine to increase Ag expression (Weber et al. 1994). More potent vaccines might potentially overcome this problem so long as epitope spreading could overcome microheterogeneity. Alternatively vaccination could be combined with checkpoint inhibitors. Further clinical evaluation will be required to assess these options.

## Evolution

The three “E’s” elimination, equilibrium and escape were postulated by Schreiber, Old and colleagues almost two decades ago (Dunn et al. 2002). Central to this was the concept of immune editing, or the sculpting of the cancer by the immune response. In addition to heterogeneity driving clonal selection, immune responses and the inflammatory characteristics of the TME can evolve and each can shape the other (Fig. 2). Indeed the immune response may co-evolve in response to the changing cancer. Loss of HLA class 1 has the potential to render tumour cells unrecognisable (Khong and Restifo 2002; Nicholaou et al. 2011). Since this is independent of tumour antigen specificity, vaccination is unlikely to be helpful in reversing this. The caveat is that Class I loss can be reversed through upregulation in response to local IFNγ. If so, incoming vaccine-generated effectors can potentially alter the cytokine milieu of the TME. Additionally, vaccination can potentially induce an immune cascade secondary to the release of antigens with consequent epitope spreading that could result in a second tier response. This has been reported where response to a melanoma-associated antigen (MAGE-3) vaccine was associated with large numbers of intratumoural T cells against another quite separate antigen MAGE-C2 (Lurquin et al. 2005). Equally, innate effectors have the potential to secrete cytokines in the TME so antibody-directed therapy against T cell surface targets have the potential to modify the inflammatory characteristics of the tumour microenvironment and potentially induce antibody-dependent T cell-mediated cytotoxicity (ADCC), and complement-dependent cytotoxicity (CDC). For instance, with antibody-directed therapy against the cell surface glycolipid ganglioside GD3 in melanoma was found to induce inflammation in tumours after systemic infusion of antibody (Scott et al. 2005).

## Inflammation

Our work and that of others have shown that the inflammatory milieu within the tumour microenvironment can have profound effects on the repertoire of antigens that are specifically presented (Woods et al. 2016b). Furthermore, studies show that IFNγ alters the proteasomal cleavage sites in defined antigens (Van den Eynde and Morel 2001). This can result in mismatch in epitope specificities between IFNγ inflamed and uninflamed conditions. Studying NY-ESO-1-specific epitopes presented on different HLA Class I molecules, we showed that that can lead to escape from T-lymphocyte killing (Woods et al. 2016a). Thus, antigens that are present in an uninflamed tumour microenvironment may no longer be present when the microenvironment became more inflamed and vice-versa. Consequently, the structural specificities required for lymphocyte recognition can come and go depending on the quality of the intratumoral inflammation. This clearly has implications for the development of peptide-specific approaches.

In summary, progressive editing and changes in the inflammatory characteristics of the TME may need to be considered when seeking to optimise immunotherapy. As a result of temporal changes, there may be completely different requirements when tumour burden is low, such as in the adjuvant setting compared to advanced disease, where editing and immune subversion will have had a far greater opportunity to shape the tumour and select resistance mechanisms. In practical terms, it may not be feasible to continue to design vaccines for individual patients in the face of continuing tumour evolution.

## An approach towards personalisation

As the field evolves, immunotherapy is likely to become more personalised. Accordingly, patients will need to be assessed using a panel of diagnostics that will help inform their likelihood of obtaining clinical benefit from one of the many immunotherapeutics available to treat their cancers. This is likely to include an assessment of tumour including the characteristics of TIL infiltrates, genomic analysis for TMB and driver mutations, the presence of biomarkers that define targetable mechanisms such as PD-L1 and potentially other immune checkpoints. Assays are also available for expression of antigens, either by sequencing, gene expression or IHC. For therapeutic vaccines to play a useful role for treating advanced cancers, an appropriate niche will need to be identified. Table 2 shows a proposed framework for evaluating this.

The TME can be evaluated to determine whether or not there has been immune engagement. Biomarkers might include the presence of effectors (a hot microenvironment), an IFNγ gene signature (Thorsson et al. 2018) or the expansion of antigen-specific T cell clones either in the tumour or in peripheral blood (Gros et al. 2016). Similarly the presence of antibodies against tumour antigens in peripheral blood clearly identifies evidence of immune engagement with tumour albeit by B cells (Beeton-Kempen et al. 2014). For those patients where an immune response has been generated but immunity has failed, the characterisation of regulatory mechanisms can enable therapy to be directed against those mechanisms that are subverting the immune response. Additionally genotyping by sequencing tumour DNA may identify molecular pathways which are contributing to T cell exclusion such as activation of the WNT/β-catenin signalling pathway (Spranger et al. 2015). Such pathways can potentially be targeted with specific drugs.

**Fig. 3:**
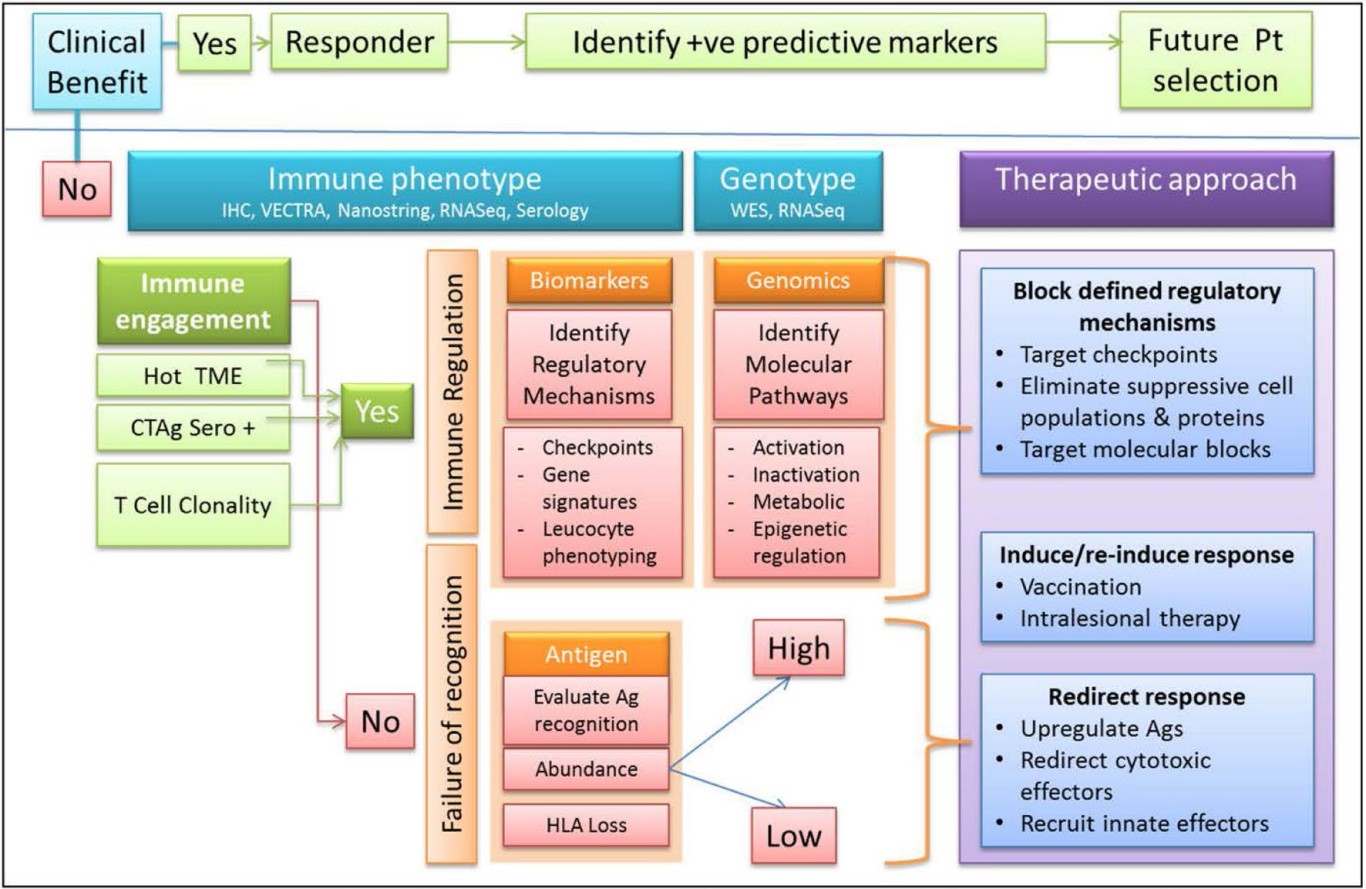
Classification and therapy approach based on integrating genotype and immune data. Hot TME: inflamed tumour microenvironment: characterised by inflammatory infiltrates. Each of the assay methods will enable further characterisation based on the density of inflammatory infiltrates and the finer immune characteristics defined by dominant cellular populations and inflammatory markers. Immune engagement: each of these assays provides an indication that the immune system has engaged with antigens within the tumour. Such Ag-specific responses can be further characterised by evaluating Ab and T cell responses using serology and TcR clonality. If antigens are present, but immune responses are absent, vaccination or intralesional therapies have the potential to induce the necessary immune engagement required. If antigens abundance is low, then approaches that upregulate antigen expression or target these with effectors such as CAR T cells may be necessary

In those cases where there has been failure of immune recognition, the role for vaccination can be considered by evaluating the tumour for the presence of antigens, presence or absence of HLA expression and then adapting an approach either with vaccination, intralesional therapy or by redirecting therapy with T cell effectors which have the potential to either eliminate the tumour or establish an inflammatory microenvironment to set the scene for subsequent spontaneous second tier immune response. The ability to reverse HLA loss will depend on the underlying mechanism, and inflammatory signals can potentially restore HLA Class I. However, if loss occurs as a result of structural genomic changes or as a result of β2M homozygous deletion, IFN-γ will not drive the re-expression of these lost alleles. Figure 3 outlines a potential clinical strategy approach that might be taken in which personalising treatment and directing patients to the therapies most likely to benefit.

## Conclusion; defining the niche for cancer vaccination

The future of oncology is an exciting one and in large part will be shaped by advances in Immuno-oncology. Of the many approaches that are currently available to treat advanced cancer with immunotherapeutics, vaccination has been historically important but has very little clinical impact. If vaccination finds a role, it will likely be as part of a rational approach aimed at defined mechanisms. Our understanding of the role of antigens and immune responses against these is now sufficiently mature for us to be able to conceive of such trials that select patients based on those characteristics most likely to benefit, and to evaluate these prospectively. The pursuit of complex approaches, such as individualised vaccines based on neo-antigen predictions, can be justified (i) if trials show that the approach succeeds where others fail and (ii) the challenges of expense and speed can be overcome. Otherwise, off-the-shelf approaches will dominate. For many these will still require personalisation; however, whether vaccination is among the options remains to be seen.
